# Molecular imaging: spawning a new melting-pot for biomedical imaging

**DOI:** 10.2349/biij.2.4.e28

**Published:** 2006-10-01

**Authors:** BJJ Abdullah

**Affiliations:** Department of Biomedical Imaging (Radiology), Faculty of Medicine, University of Malaya, Kuala Lumpur, Malaysia

**Keywords:** Molecular imaging, genomics, proteomics, diagnosis, treatment

## Abstract

Predicting the future is a dangerous undertaking at best, and not meant for the faint-hearted. However, viewing the advances in molecular medicine, genomics and proteomics, it is easy to comprehend those who believe that molecular imaging methods will open up new vistas for medical imaging. The knock on effect will impact our capacity to diagnose and treat diseases. Anatomically detectable abnormalities, which have historically been the basis of the practice of radiology, will soon be replaced by molecular imaging methods that will reflect the under expression or over expression of certain genes which occur in almost every disease. Molecular imaging can then be resorted to so that early diagnosis and characterisation of disease can offer improved specificity. Given the growing importance of molecular medicine, imagers will find it profitable to educate themselves on molecular targeting, molecular therapeutics and the role of imaging in both areas.

While medicine has taken on a molecular character and the Human Genome Project has yielded a wealth of information for mapping the human body, nothing really explains how the body works. Molecular imaging will play a key role in delivering molecular medicine since it defines many cellular and biochemical mechanisms [1]. It must be said that although the term “molecular imaging” is relatively new, the underlying concept has been the basis of many nuclear medicine procedures for over half a century. In fact the imaging of biological processes is central to the scientific method in natural sciences [2]. The term molecular imaging can be broadly defined as the “visualisation and characterisation of biologic processes at the cellular and molecular level *in vivo*”. And this has been primarily catalysed by two concurrent developments, namely the understanding of the molecular basis of disease, and the development of drugs directed at these molecular targets.

Molecular imaging is a growing research discipline (and increasingly a clinical discipline too) aimed at developing and testing novel tools, reagents, and methods to image specific molecular pathways with whole body imaging instruments *in vivo*. The pathways targeted play a key role in disease processes. Molecular imaging holds the unique potential of being able to find, diagnose and treat disease *in vivo* simultaneously (i.e., inside the body), as well as depict how well a particular treatment is working i.e., theragnostics. This is in contrast to “classical” diagnostic imaging where most of diseases are diagnosed based on the manifestation of signs and symptoms following which remedial therapy is then applied to modify or ameliorate those symptoms. In most diseases, since the origins are often unknown and even in those instances where it is known, the treatments are meant at best to keep things under control.

Molecular imaging exploits specific molecules as the source of image contrast. This paradigm shift from non-specific physical to specific molecular sources underlies many of the current molecular imaging research efforts. It is this search for the “holy grail” of modern medicine that may make it possible eventually to manipulate an individual's genetic constitution appropriately, to get rid of disease processes.

Molecular imaging is to a certain extent technology driven since molecular information can be obtained with some “high-end” imaging technologies. To do more would require newer equipment. However, on a conceptual level, molecular imaging is neither about technology nor about a change in the practice but essentially another revolution in the way medicine is viewed. One underlying premise of molecular imaging is that this emerging field is not defined by the imaging technologies that underpin acquisition of the final image per se but rather by the underlying biological questions of medicine. The ramifications will be felt in every nook and cranny of medical practice and then extend into the legal, social and ethical aspects of society. No concept and basis of medicine, currently practiced will be spared.

As a consequence, a new approach to wellness, disease prevention and treatment needs to be developed to counter the effects of poor health and illness, especially in the world’s less developed countries. To enhance such an approach, the potential contributions of biomedical imaging, bioengineering, and bioinformatics to emerging research areas, such as functional genomics, proteomics, molecular biomechanics and drug delivery systems, tissue and cell engineering, quantitative biology and computer modelling, molecular and computational imaging, computer-aided diagnosis, metabolic imaging, ultra fast and integrated imaging systems are of prime importance. In the current phase of the increasingly complex and ever changing health care environment, governments, medical speciality organisations, researchers, vendors, and providers are finding it hard to cope with these challenges alone. Partnerships are critical to face any new concept. It is therefore imperative that new relationships sharing the risk of adopting new technological innovation, programmes, services and processes designed to improve overall patient care be worked at.

Increasingly, the power of molecular imaging comes from the capacity to harvest vast amounts of knowledge gained from molecular biology. Currently we can theoretically design a molecular imaging agent to target each step in the sequence from DNA replication to protein synthesis and subsequently all the steps of protein metabolism. To begin investigating molecular signals *in vivo*, researchers have already developed and characterised methods for imaging endogenous proteins, such as receptors, transporters, or enzymes and their respective functions. More recently, methods have also been validated to analyse specific expression of transgenes of interest. This is typically performed by quantifying the activity of reporter genes delivered by a viral vector, as used in gene therapy, or through use of exogenous reporter genes in genetically engineered cells [3] and transgenic mouse models [4].

Processes in the human body are extremely complex and depend on a multitude of factors. As such, they are much more difficult to analyse than a laboratory sample. In fact, metabolic processes can only be truly understood *in vivo*. If we could improve the analysis of metabolic reactions in living organisms, it would lead to immense savings for the pharmaceutical companies and open a promising market. Methods that produce visual images are being used with increasing frequency to study metabolic processes, e.g., to find out when individual genes become active (are expressed). Here, highly specific molecules (probes) are employed, which often are customised with genetic engineering. These special molecules search for a specific substance and, when they have found it, link with it and emit signals that can be depicted visually to provide a diagnosis of changes to a patient's metabolism. The key elements to sampling molecular information are [5]:

- The use of special imaging probes with high specificity. In fact due to the advances in chemistry and screening it is possible to have increased specificity without sacrificing sensitivity.
- The availability of appropriate amplification strategies
- High resolution images from systems with increased sensitivity, and
- Capability of overcoming biological barriers to delivery of probes

With regard to the strategy for imaging these probes:

- Direct imaging uses a probe specific for cell surface receptors, intracellular molecules or gene expression, which interacts directly with the target providing an image intensity correlating to the amount of target actively present.
- Indirect imaging is more complicated as it often uses both a reporter gene and a reporter probe which interacts within specifically targeted cells to produce a metabolite trapped in the cells that is visualised when scanned.
- Surrogate imaging detects downstream effects of endogenous molecular-genetic processes using established radiopharmaceuticals and clinical imaging protocols.

Regarding the imaging probes there are three basic types [6]:

- The compartmental probe typically assesses physiological parameters (*i.e.*, flow and perfusion) and as mentioned above the probe does not directly image the molecular process but a surrogate.
- Targeted probes act directly against a specific moiety targeted to the molecule, receptor or enzyme of interest or an imaging component that provides the physical contrast.
- Finally, “smart” probes activate exclusively in the presence of their intended target and since there is no significant background signal, smart probes have a significant signal advantage over simple targeted agents. Primarily because probes need to be biocompatible, the presence of additional delivery barriers [7], and the necessity for developing special *in vivo* amplification strategies [8], *in vivo* molecular imaging is more challenging than in vitro detection.

Optical imaging technology (including diffuse optical tomography, phase-array detection, photon counting, near-infrared fluorescence imaging), high-spatial-resolution MR and nuclear imaging techniques (e.g., positron emission tomography [PET]), fusion imaging and micro imaging systems (micro CT, micro MR, micro US) play an important role in the field. Each of these techniques has its particular advantages and disadvantages, and the use of one or the other technique is mostly dependent on the specific research question and hypothesis to be tested [2].

Radionuclide imaging devices visualise very low concentrations of radionuclide probes (nano- to femtomolar) in real-time [8] and provide quantitative information [9], but with low image resolution. They can be used for whole body imaging. PET is frequently used when a substrate to a given target exists that can easily be labelled with a positron emitter, for example labelled 2′-fluoro-5-iodovinyl-1-ß-D-arabinofuranosyl-uracil (FIAU) or ganciclovir for imaging of viral thymidine kinase gene expression. Radio nucleotide imaging combined with a computed tomography (CT) or a magnetic resonance imaging (MRI) scan provides high anatomic definition along with functional imaging for precise location of the selected molecular activity. Nuclear imaging techniques are suited to track small amounts of labelled therapeutic drugs, and to investigate multiple drug resistance, or delivery systems such as viral vectors. However the downside is the need for a cyclotron and high cost.

Magnetic Resonance Imaging's (MRI) ability was enhanced significantly with the development of functional MRI. The revival of interest in molecular imaging has expanded the frontiers of MRI even further. MR imaging has two particular advantages over techniques that involved the use of isotopes, namely the higher spatial resolution (micrometer rather than several millimetres), and the simultaneous extraction of physiologic and anatomic information.

In comparison with isotope techniques, however, MR imaging is several magnitudes less sensitive (millimolar rather than picomolar), which is why reliable signal amplification strategies must be developed. MR techniques in cell imaging are also maturing where cells are induced to take-up superparamagnetic iron oxide formulations [10].

Ultrasound in molecular imaging allows real-time imaging with resolution of less than 50 µm. With the developments of microbubble technology [11] and harmonic imaging, ultrasound is increasingly being used to translate molecular processes *in vivo*. Even though CT is often not recognised as a modality for molecular imaging, it has a role especially for the study of soft tissue tumours in bone and lung because of its low cost, reasonable resolution and fast scanning times.

Finally, optical imaging techniques have already been developed for applications in molecular and cellular biology (e.g., fluorescence microscopy) and *in vivo* surface imaging [12]. One of the appealing advantages of near-infrared optical imaging is that quenched fluorescent labels that become brightly fluorescent after specific molecular interactions with their targets, can be used.

Another notable advantage of optical techniques is multiple probes with different spectral characteristics can be used for multichannel imaging, similar to in vitro karyotyping [3]. In addition, it is reasonably cheap, has good spatial resolution and possesses nanomolar sensitivity. Newer approaches have been advocated that may ultimately lead to the development of tomographic optical imaging systems in the near-infrared spectrum. These have been suggested to overcome depth penetration.

Today's scientists are not just looking at specific molecules, but are also evaluating the components within the nucleus of their cells. They are learning what causes the cells to turn on and off, what makes them do what they do and how their fundamental function can be boosted or shut down. While molecular imaging has significantly advanced oncology, cardiology, neurology, infectious disease detection and therapy, drug development, and disease treatment, even more is expected. Here is what molecular imaging promises:

## Detecting disease

In the future, advances in molecular imaging will lead to the development of a broader array of imaging probes that will cover all the body's major systems and associated disease types, making even earlier detection of disease possible. Our capacity to image these molecular changes will directly affect patient care by allowing much earlier detection of diseases, e.g. image molecular changes that we currently define as “predisease states”. If such a situation came to pass, then we would allow intervention when the outcome can be significantly altered. Because of the greater specificity of the imaging probes and techniques we would be able to look for specific cell types, e.g., cancer cells have an increase in metabolic activity in comparison with normal cells. This fact makes it possible to image cancer cells *in vivo* using deoxyglucose, a metabolic substance that is voraciously glycolised and trapped by targeted cancer cells. By labelling deoxyglucose with a radioactive agent and injecting the resulting molecular imaging agent into patients, scientists can make nuclear images of the primary tumour as well as metastatic sites throughout the body.

When a cell is dying (apoptosis), it turns inside out, presenting an otherwise unexposed protein binding site. The body responds by producing a protein called annexin, which seeks out and connects to the binding site of these dying cells to “tag” them for destruction by the immune system. By creating a human annexin, attaching it to the imaging agent technetium and injecting it into the patient, scientists can “seek and illuminate” dying cells. Physicians can use this information to decide whether to change or keep a patient's therapy regimen. For example, when chemotherapy or radiotherapy is used to kill cancer cells, apoptosis occurs. Apoptosis is the necessary death of cells to make way for new cells and to remove cells where DNA has been damaged to the point at which cancerous change is likely to occur. If the chosen therapy is effective, apoptosis can be demonstrated within 24 to 48 hours. Physicians can use molecular imaging to determine whether apoptosis has occurred and can change the therapy if it does not. Not only does this ability mean that the treatments used will be more effective, but further costs associated with ineffective therapy can be avoided. This can be done using SPECT (single photon emission computed tomography) or MRI. In the example quoted above if therapy is proving effective, then, annexin could be used as a delivery vehicle to further enhance cell death. Physicians would add a payload of radioactive toxin to annexin. When injected, the “loaded” annexin will deliver the toxic agent to the site of the dying cancer cells to cause even more cells to die. This process creates a cycle of cell death because the more cells that die, the more toxin-loaded annexin will be attracted to the cancer site. This molecular chain of events helps accelerate the therapy's effectiveness.

With the US Food and Drug Administration's (FDA) approval of Avastin (Bevacizumab; Genentech, Inc., San Francisco, CA) as a first-line treatment for patients with metastatic colorectal cancer, it was a major landmark in the field of angiogenesis. With the development of an image based angiogenetic marker, it would help select patients for appropriate therapeutic regimens including the most appropriate combination of angiogenic and other therapeutic agents, help to identify the optimal time window and the appropriate dosage of the different therapeutic agents, assist in monitoring the effects of such treatments, and provide functional information for adjusting the therapeutic regimens over time in an interactive basis [13]. Since imaging can potentially provide morphologic, functional and molecular information in a spatially and temporally resolved manner, many investigators have incorporated imaging into pre-clinical studies and clinical trials of angiogenesis therapies.

Further developments are occurring where imaging and therapeutic agents are tagged with a targeted molecular agent are being developed and this may enhance treatment e.g. Zevalin (Ibritumomab tiuxetan, IDEC Pharmaceuticals, Cambridge, MA), a therapeutic regimen for treatment of relapsed or refractory low grade, follicular or transformed B-cell non-Hodgkin's lymphoma. Indium-111 labelled Zevalin scanning allows visualisation of disease and calculation of dose to be delivered by Yittrium-90 labelled anti-CD 20 monoclonal antibodies [14].

Current evidence suggests that one course of anti-CD20 radio immunotherapy is as efficacious as six to eight cycles of combination chemotherapy.

Decreased tissue oxygen tension is a component of many diseases. Although hypoxia can be secondary to a low inspired P0_2_ or a variety of lung disorders, the commonest cause is ischemia due to an oxygen demand greater than the local oxygen supply. In tumours, low tissue p0_2_ is often observed, most often due to a blood supply inadequate to meet the tumour's demands. Decreased tissue oxygen tension is a component of many diseases. In the heart tissue hypoxia is often observed in persistent low-flow states, such as hibernating myocardium. In patients with stroke, hypoxia has been associated with the penumbral region, where an intervention could preserve function. In some tumours the efficacy of conventional radiotherapy is limited by the presence of a hypoxic, radioresistant, and repair-proficient subset of tumour cells.

Despite the potential importance of oxygen levels in tissue, difficulty in making this measurement *in vivo* has limited its role in clinical decision making. This has led to the development and testing of hypoxic imaging techniques and agents. An ideal hypoxia imaging agent should have high membrane permeability for easy access to intracellular mitochondria and low redox potential to confer stability in normal tissue, but it should be able to be reduced by mitochondria with abnormally high electron concentrations in hypoxic cells. Imaging with some of these agents can provide direct evidence of tissue with low oxygen levels that is viable. In the experimental setting this information is useful to plan a more aggressive approach to treating tumours, or revascularise a heart suffering ischemic dysfunction. Oxygen electrode measurements in animal experiments have demonstrated a strong correlation between low tumour pO_2_ and excess ^60^Cu-diacetyl-bis(N(4)-methylthiosemicarbazone (^60^Cu-ATSM) accumulation. Some studies have suggested that pretreatment imaging with ^18^F-2-fluoro-2-deoxy-D-glucose(FDG) and ^64^Cu-ATSM would allow stratification of tumour phenotypes such that those tumours that are most susceptible would be selected for treatment [15].

## Aiding and assessing response to therapy

Currently treatment instituted for most diseases is based on “one size fits all”. This is based on our inability to separate the responders from those who are either not likely to respond or those that may actually develop more complications from the treatment offered. But increasingly many new-generation therapeutic drugs are designed with highly specific molecular targeting capabilities and delivery mechanisms. By directly imaging the underlying alterations of diseases, we have the potential to be able to directly image the effects of therapy. This would provide an opportunity to play a direct role in determining the efficacy of treatment. Further, response to therapy could be assessed shortly after therapy has been initiated and not in the many months subsequently as required today to determine whether pharmacological or biological intervention has been beneficial.

In addition the toxic effects of treatment on the patient's healthy tissue can be avoided. In the future, molecular imaging is expected to aid in identifying the presence of drug-resistant genes that will enable clinicians to pre-determine which treatment regimens will be most effective within an hour of initiating treatment and avoid delays in optimizing a patient's therapy.

Furthermore, molecular tracers, or radiopharmaceuticals, will be used to create diagnostic images which visually indicate whether cancer patients are susceptible to multi-drug resistance, a condition in which the defensive response to one type of chemotherapy also diminishes the potency of other chemo agents. With this information, physicians can make accurate and fairly rapid diagnoses and provide patients with more precise treatment.

Others are working on nanoparticle delivery platforms that can potentially deliver both imaging and therapeutic agents to endothelial targets. Using a vascular targeted imaging agent for selecting patients to be treated and for monitoring response with the therapeutic agents delivered via the same delivery vehicle satisfies the requirements for “personalised treatment” [16-18].

## Enabling nano-treatments

It may seem like the movie, Osmosis Jones (Warner Bros) where researchers are working to integrate molecular imaging with nanotechnologies (human-made, molecular-size structures or machines) to detect disease and enable even more precise therapy in many instances eliminating if not minimising the need for surgery entirely. Some of envisioned scenarios may include the development of biosensors providing the exact location of disease, and new nanotechnologies which can be dispatched into the body that can deliver drug therapies directly to cancerous cells by affixing themselves to those specific cells and releasing cancer-killing agents.

Just like nano-robots, these nanotechnologies shall be designed to self-assemble themselves at the appropriate place and time following which they are able to repair bones or tears and if necessary stimulate the growth of new blood vessels or tissue. There would additionally be imaging technologies which could monitor the process, ensure that it is working properly, and measure the results, the ultimate of image guided minimally invasive surgery!

## Drug discovery cycle

Molecular imaging will facilitate the development of new drugs, by providing early stage chemical compounds that will enable researchers in the public and private sectors to validate new drug targets, which could then move into the drug-development pipeline. This is particularly true for rare diseases, which may not be attractive for development by the private sector. Three key technological advances drive NIH's effort to build small molecule libraries.

- The successful completion of the Human Genome Project has provided an enormous cache of human biology to be studied and potential drug targets to be discovered.
- Developments in chemistry have given researchers in the public sector the ability to synthesise large numbers of related molecules, a capability previously available only to researchers in pharmaceutical and biotechnology companies.
- Advances in robotic technology and informatics now allow scientists to screen hundreds of thousands of compounds in a single day, an orders of magnitude greater capacity than was available a decade ago [19].

Molecular imaging is a powerful concept that envisions the promise of disease characterisation / phenotyping and early assessment of therapeutic efficacy. From this, it is a logical step to envisaging “personalised medicine,” where disease phenotyping will be used to tailor the most optimum therapeutic regimen patient by patient and not the usual one size fits all. This is already happening but is not directly related. Imaging entails the use of Her-2/neu gene expression as an indicator of whether breast cancer patients will respond to the drug Herceptin, a monoclonal antibody.

Today the power of imaging is so sophisticated that we now have the capacity to identify unresolved biological and clinical questions and focus on how imaging techniques might be used to solve these problems. Emphasis continues to be on minimising invasiveness, reducing image processing time, lower cost, less radiation dose but at the same time maximising resolution and contrast as well as easy interpretation of data. Future advancements lie in not only in new modalities e.g. optical imaging but also in improved imaging modalities as well in producing displays that intelligently combine structural, chemical, electrical, magnetic, acoustic, and motion information.

However though in the past, diagnostic imaging tests were designed to be stand alone investigations with specialised image acquisition, analysis and display, it is important that we do not continue to utilise diagnostic imaging tests in this same manner, but rather exploit the synergies between each individual i.e. the sum of the parts is larger than each alone! For example, the development of PET/CT systems allows the synthesis of the rich metabolic and functional information gained from PET with the morphologic information provided by CT, new information can be gained that cannot be obtained with each modality alone. These multi-modality systems (together or image fusion from two separate systems) is only the beginning of what may prove to be a significant paradigm shift in medical imaging device design and manufacturing [20].

Molecular imaging promises to become a powerful addition to the ammunition of medical imaging. “Who controls and gives direction to that growth remains arguably an open question. Today’s imaging research defines the practice of the future” [2]. Leaders in imaging generally will be well served to acquire the knowledge necessary to incorporate these new methods into their practices, including knowledge of fundamental principles in molecular biology. They should also promote education and research that will undoubtedly impact the future of imaging.

The 21st century will witness further innovation, growth and clinical utility imaging and therapy. These challenges are already formidable enough for imaging community in the developed world but be daunting for those in the developing countries where meeting the current imaging needs is at most times, barely sufficient. Despite these challenges, we in the Southeast Asian region must make a concerted and co-ordinated effort to ensure that we are not left behind. The following are some of the actions that will help ensure that the imaging community here does not lag behind.

Firstly, leaders in imaging generally should recognise the importance of this new and rapidly expanding field. They need to encourage acquisition of knowledge necessary to incorporate these new methods into their practices, including knowledge of fundamental principles in molecular biology. They should also promote education and research that will undoubtedly impact the future of imaging.

Although we may not be able to change the current line of thinking of the current practitioners; the future generations however, must be aware of the developments in molecular imaging and so there must be changes in the curriculum with introduction of subjects like biochemistry, immunology, as well as oncology. This is being incorporated in the new integrated curriculum implemented in some institutions.

Thirdly, there should be greater co-operation as well as coordination between the regional societies, e.g., ASEAN Association of Radiology and Asian-Oceania Society of Radiology, in promoting greater awareness of biomolecular imaging. This is being done by the introduction of specific seminars and conferences [21], and would certainly go a long way in ensuring greater awareness and interest. Greater co-operation with other regional societies, e.g., the European Congress of Radiology (ECR), will also facilitate this process with the availability of online teaching materials [22] from the conferences.

Finally, the setting up of local, national or regional centres of excellence in biomolecular imaging would be another initiative to ensure and maintain the lead. These centres are also important since the disease patterns and demographics are very different from that in the West. However, setting up such centres is intensive: both financially and in terms of human resource and is probably beyond the reach of many countries but Singapore [23], Thailand and Malaysia would surely be able to do so if they have not already done so. Such centres should be involved in the training of the new generation of imaging specialists and should provide for cross training and fellowships.

This potential requires interdisciplinary partnerships and collaborative efforts between physicians, medical physicists, biomedical engineers and computer scientists. We will all need to learn each others' technical language to move forward. The setting up of a regional journal such as biij (www.biij.org) is also essential in putting all these different aspects together. It also provides a forum for publication, dissemination, and discussion as well as networking between the different groups and organisations in the region.

In view of these changes, as well as the increasing emphasis on imaging at the cellular level or even at the genetic level, research in the field of biomedical imaging is set to explode.

Impossible is nothing!
